# Evaluating the impact of land use and land cover changes on forest ecosystem service values using landsat dataset in the Atwima Nwabiagya North, Ghana

**DOI:** 10.1016/j.heliyon.2023.e21736

**Published:** 2023-10-28

**Authors:** Richard Baidoo, Kwame Obeng

**Affiliations:** Department of Geomatic Engineering, Kwame Nkrumah University of Science and Technology, Kumasi, Ghana

**Keywords:** Land use, Land cover, Remote sensing, GIS, Forest ecosystem service values

## Abstract

This study investigated land use and land cover (LULC) changes and its impact on forest ecosystem service values for 20 years in the Atwima Nwabaiagya North District using Landsat images of 2002, 2012 and 2022. Supervised classification with Maximum Likelihood Algorithm was used to classify the Landsat images. Five LULC types (high-dense forest, low-dense forest, water, bare-ground, and Built-up area) were successfully classified, with overall accuracies of 99.0 % and Kappa coefficients of 0.99. The result of the study showed a reduction of high-dense forest to 23.87 %, low-dense forest to 26.53 %, and water areas as 1.16 % whereas built-up (21.44 %) and bare-ground (27 %) experienced an expansion in their land areas. Related literatures and ecological assets value table with adjusted price value were used to evaluate ecosystem service values in response to LULC changes. The study discovered that ecosystem service value for high and low-dense forests have declined from USD 22.68 million and USD 8.75 million to USD 14.56 million and USD 5.2 million respectively. The overall total ecosystem service value declined by USD 33.73 million in 2002 to USD 21.91 million in 2022. It was revealed that the most notable feature to changes in forest ecosystem service values was the expansion of built-up and bare-grounds. There is a need to curb the current drivers of LULC changes in the Atwima Nwabiagya North to stop further forest degradation for optimum delivery of forest ecosystem service values in the district. For land use planners and decision makers who need site-specific information on the effects of LULC alterations on values of forest ecosystem services, the study's findings are essential. This will make it easier to track past environmental changes and obtain quick, accurate results for use in decision-making.

## 1 Introduction

Forests, which are abundant in resources cover one-third of the earth's land area [1]. These resources include food, timber, and fiber amongst others [2]. As human population continue to increase [3], so as urban development grows and demand for natural resources [4]. These have led to a major threat to the integrity of ecosystems [5] affecting their biophysical structure, taxonomic and functional diversity, and ecological processes [6]. Studies have revealed that interactions between nature and human activity are major variables influencing the amount of forest cover in many places of the world [7,8]. Human impact on nature's ability to create and protect vital essential abiotic and biotic resources are consequences of LULC changes [[9], [10], [11]].

Land use refers to how humans use the land while land cover refers to the physical and biological state of land [12]. Forest ecosystems are regions of the landscape where trees predominate. They are made up of biologically integrated communities of plants, animals, and microorganisms, as well as the nearby soils (substrates) and atmospheres (climates) that they interact with [13]. Forest ecosystems provide specialized services of different quality and quantity [14]. These include ecotourism, recreation, and provision of habitat for biodiversity, preservation of soil, carbon sequestration, resource availability, and water quality [15]. Provisioning (i.e., food and water), regulating (i.e., management of climate and disease), supporting (i.e., nutrient cycles and crop pollination), and cultural (i.e., spiritual and recreational advantages) are the services provided by ecosystems [16]. Recognizing these services makes a significant contribution by reframing how people and nature interact in ways that promote biodiversity preservation, ecosystem management, and sustainable development [17]. Despite the enormous contribution forests provide to the sustainability of human well-being and survival, they have suffered substantially from global deterioration [[18], [19], [20]].

Studies on Land use and Land cover (LULC) have recognized the serious risk that LULC change poses to socio-ecological systems on a global scale [21]. These have raised concern regarding the functions and processes of ecosystems [22,23], especially in developing countries where majority of the tropical forest on earth is found [24]. The natural environment has suffered as a result of cities' sprawling into peri-urban areas. This has hampered and altered regional ecosystem processes, biogeochemical cycles, and climate [25]. Due to the relocating of business and residential activities into rural areas on the outskirts of metropolitan centres, arable lands have been lost [26,27]. A study by Costanza et al. [28] has estimated a loss of ecosystems services worth of $4.3–20.2 trillion every year since human societies derive essential benefits from the natural ecosystems [29]. This is not uncommon in the Atwima Nwabiagya North District where majority of wet, forest and agricultural lands have been affected. The Atwima Nwabaigya North District is quickly transitioning from rural to urban with many forested lands turned into settlements and other land uses [30]. These have been linked to a variety of human activities [31], due to its proximity to the Kumasi Metropolitan Assembly [5]. The Atwima Nwabiagya North district contains three important tourist sites i.e. Owabi Wildlife Sanctuary (serving as a habitat for various plant and animal species), and Owabi and Barekese watersheds which supply water to residents of the district and Kumasi as a whole [32].

However, inimical human activities have affected the forest cover [30], Owabi reservoir [33], socioeconomic factors [34] livelihoods [5] and biological diversity [31]. These have resulted in the potential reduction of some of the ecosystem services provided by the Owabi wetlands and wildlife sanctuary within the district [16]. Studies have been done to investigate how encroachments have affected ecosystem services within the district [16,35]. However, these studies were primarily focused on the use of interviews and household structured questionnaires, with no emphasis on the application of remote sensing and Geographic Information Systems (GIS). For monitoring, mapping, and evaluating LULC changes over time at various geographical and temporal resolutions, many studies have made use of various satellite imagery [36]. Long-term spatial data archives are made available by satellite images for ecological assessment, monitoring, and management [37]. They are employed in a variety of studies, including those on the provision of ecosystem services, the monitoring and mapping of wetland extent, the calculation and mapping of biomass, the use of soil moisture, inundation mapping, and the monitoring of water level [[38], [39], [40], [41], [42], [43], [44], [45]].

Remote sensing and GIS tools have opened new areas for ecosystem research [46], providing time sequence data of LULC and makes it possible to quickly acquire data at a price that is equivalent to ground survey methods [ [25,47]]. They provide established, affordable methods for comprehending the dynamics of landscape [34,48] beneficial for natural resource management, monitoring, and mapping [[49], [50], [51]]. They have presented intriguing findings and provided some crucial policy recommendations for ecological land management [52,53]. Due to these, its potential impacts on the environment at the local, national, and international levels are taken into consideration [54,55]. Therefore, this study fills a research gap by utilizing remote sensing, and GIS tools to investigate the extent to which LULC changes have affected forest ecosystem service values in the Atwima Nwabiagya North district. The aim of the study is to: determine LULC change impacts on forest ecosystem service values in the Atwima Nwabiagya North District. The objectives of the study are to.

1. Investigate LULC changes in the Atwima Nwabiagya North district from 2002 to 2022.
2. Assess the current state of forest ecosystem service values.
3. Examine LULC change and its impact on forest ecosystem service values.

## 2 Materials and method

### 2.1 Description of the study area

**Fig. 1:**
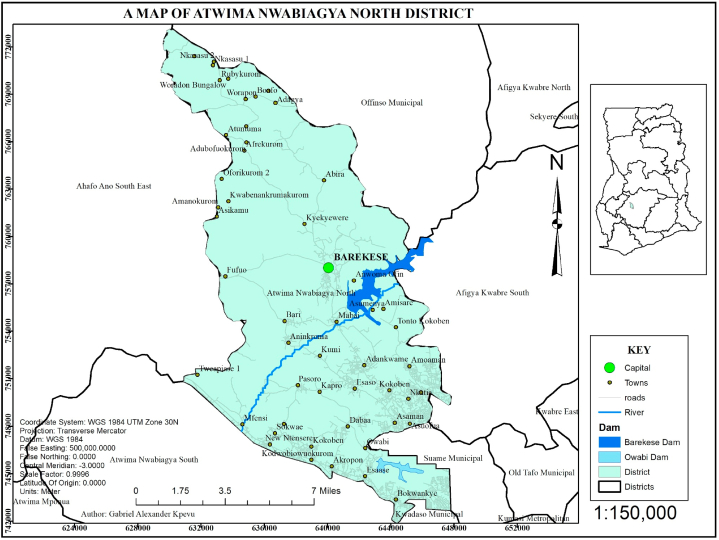
Map of the study area.

**Fig. 2:**
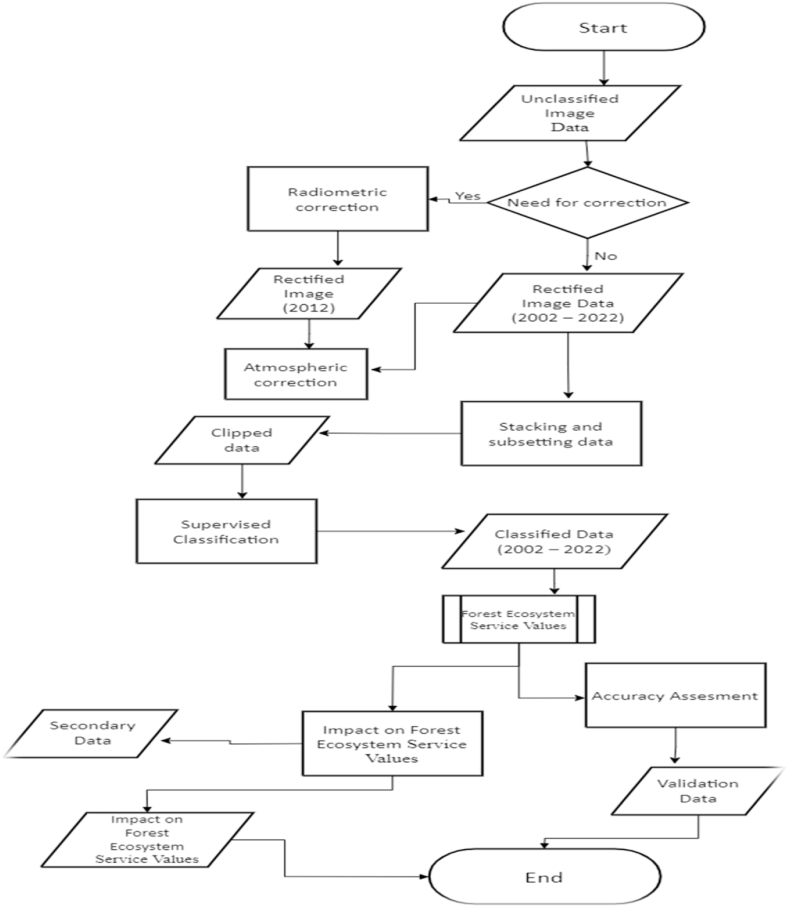
Flow chart of activities.

The Atwima Nwabiagya North District is situated in the Ashanti region, Kumasi, Ghana. It lies between latitudes 6° 47' 42.7" and 6° 42' 6" North and longitudes 1° 43' 16.8" and 1° 35' 29.4" West of Kumasi in Ashanti region [56], it is roughly 19 km North West of Kumasi [56]. The study area is bounded to the North by the Municipality of Ofinso, and Afigya Kwabre North, to the South by the districts of Kumasi Metropolitan Assembly and Kwadaso Municipal, to the East by the districts of Afigya Kwabre South, Old Tafo Municipal and Kwabre East, and to the West by the district of Ahafo Ano South East, Atwima Mponua, and Atwima Nwabiagya South (see Fig. 1), lying approximately 23 km northwest of Kumasi [16,35]. The district contains the Owabi and Barekese catchments [31,56]. It is a section of the region with a humid semi-arid climate, with double maximum rainfall seasons averaging 170–185 mm annually [57]. Rain does not fall in a predictable pattern throughout the year [5]. Temperatures are quite consistent, ranging from 27 °C in August to 31 °C in March [57]. Human activities have severely disrupted the vegetation, depriving it of significant plant and animal species as well as other forest products [5] (see Fig. 2).

### 2.2 Field data collection

The data used for the study were collected through field observation, related literatures and Landsat images of 2002, 2012 and 2022 of the study area. This time frame was chosen because it was suitable for identifying forestland from other types of land cover in the study area. The images were chosen based on their accessibility, spatial resolution, and general quality, particularly those with little cloud or scene cover [58]. In order to capture LULC feature coordinates within the study area, the Global Positioning System (GPS) and Google Earth were utilized to gather point data in geographic coordinates from field surveys. 100 sample points in total were chosen at random and are shown in Table 6, Table 7, Table 8. These sample points served as the basis for testing the accuracy of the classified images and validating the LULC classification [59].

### 2.3 Remote sensing data processing and analysis

LULC and change dynamics of the study area were investigated using Landsat data from the United States Geological Survey (USGS) for the years 2002, 2012, and 2022. The images were cloud-free and resampled to a 30*30 m spatial resolution and were taken in the same season [8,38,60,61]. Using QGIS 3.12.5, all processing and post-classification processes were completed. Image preprocessing, which included atmospheric and radiometric corrections, was done before interpretation. By removing impurities, these changes were made to the remote sensing image to enhance its quality and readability [59,[62], [63], [64]]]. Following image preprocessing, a supervised classification technique using maximum likelihood algorithms that assign each pixel to the class with the highest likelihood were used to prepare LULC maps for the study [65,66].

To identify LULC classes in the Landsat images, band combinations, visual interpretation, GPS, and Google Earth were all used [31]. Due to their spatial resolution (60 and 120 m) and relevance for the detection of atmospheric characteristics, the bands 1 (coastal aerosol), 6 and 7 (thermal band), 9 (water vapour), 8 (panchromatic), 9 (SWIR - cirrus), 10 (LWIR-1) and 11 (LWIR-2) were removed from the study [59,67,68]. These have been viewed as being inapplicable for monitoring vegetation [69]. However, Blue, Green, Red, NIR and SWIR 1 and 2 bands were used in this study. These bands are the very popular band combination used for vegetation analysis and vegetation studies and are useful for studying various stages of plants growth [70].

The images were adjusted geometrically and projected to zone 30 N of the Universal Transverse Mercator (UTM) [38]. A shape-file of the district was used to sub-set the area of interest from the Landsat images. Using field observation, previous related works of the research area and image classification, five land cover classes i.e., high-dense forest, low-dense forest, built-up, bare-ground and water were derived for the analyses of the study. In accordance with the ideas of “complete consistency” and “temporal stability” [71,72] sample points from the five land cover classifications were filtered [71].

### 2.4 Change detection analysis and post classification

Techniques for change detection and accuracy evaluation are included in this step. The study area's LULC was monitored using a change detection approach [73]. A pixel-based comparison was used to collect change information on a per-pixel basis and to more quickly examine the changes using the "-from, -to” information [62]. This strategy is the only one that permits “from” and “to” classes to be evaluated for each modified pixel and enables the comparison of differences across independently classified images from each of the relevant years [62]. Cross-tabulation was used to compare classified picture pairs from two separate decade data sets in order to assess the qualitative and quantitative aspects of the changes for the years 2002–2022. With the help of QGIS 3.12.2 software, a change matrix [74] was created. Between 2002 and 2022, quantitative areal data on changes in LULC, as well as gains and losses in each category, were gathered. To confirm the accuracy of the classified images, ground truth points were acquired during a field study using GPS and Google Earth. Using a sample of 100 ground control points gathered using GPS and Google Earth for each year, the study verified the categorized images. This was achieved by calculating and assessing the accuracy and Kappa statistic of each classified image [75,76].

### 2.5 Classification accuracy assessment

The classification accuracies of the resulting LULC change maps for the years 2002, 2012, and 2022 were evaluated. The classified maps were validated using 100 ground control point field data samples. An error matrix was used to assess the classification process accuracy in relation to the reference data, including overall, user, and producer correctness. Along with providing evidence of how the classification mistakes occurred, the error matrix also offered a complete examination of the agreement, omission, and commission between the classification results and training data [77]. Table 6, Table 7, Table 8 show the kappa coefficients and overall levels of accuracy achieved to validate the classification accuracies. The kappa values for all the periods reflect almost perfect reliability because they range from 0.81 to 1 [78] (see Table 1).

**Table 1:** Landsat data used for the study.

Landsat Products	Acquisition date	Path/Row	Spatial Resolution	Source
Landsat TM7	31/12/2002	192/056	30 m	USGS^a^
Landsat 7 ETM+	31/12/2012	192/056	30 m	USGS
Landsat 8 OLI_TIRS	31/12/2022	192/056	30 m	USGS

a United States Geological Survey.

### 2.6 Ecosystem service value assessment

Estimating the value of ecosystem services can be done using a variety of direct and indirect techniques [79]. Each ecosystem service's value per unit area was calculated by Costanza et al. [80]. In order to calculate the changes in ecosystem service value, the value transfer valuation method was applied [81]. Xie et al. [82] estimated the ecosystem service values per unit area of the different land types based on Costanza's global ecosystem service values [83]. A modified benefit transfer approach was utilized to assess how ecosystem service value (ESV) changes in response to LULC modification on ecosystem service values. The local modified coefficient values (USD ha^−1^ yr^−1^) were used to evaluate relative losses or gains in ecosystem service value due to LULC changes [84]. Kindu et al. [84] designed ecosystem service value for 11 biomes depending on the Costanza et al. [80], method considering local Ethiopian conditions [71]. Other studies have also used updated monetary value calculated by Constanza et al., [85]. For this study, five LULC classes were chosen as a suitable proxy for LULC types: (1) cropland for low-dense forest, (2) tropical forest for high-dense forest, (3) bare-land for bare-ground, (4) Built-up for the built-up, and (5) waterbodies for water. Shrestha et al. [79], used the modified value calculated by Xie et al., [82]. As the Atwima Nwabiagya North district shares similar relief features with Xie et al. [82], the study adopted Xie et al.'s adjusted price value coefficient of the ecosystem which is shown in Table 2.

**Table 2:** Ecosystem Service Value (ESV) coefficient for different land cover types given by Ref. [82].

Land Cover Types	ESV (USD/ha/year)
Forest cover	2168.84
Bare-lands	0.00
Crop land	699.37
Waterbodies	6552.97
Built-up	0.00

The ecosystem service value is calculated for the identified five (5) LULC classes, which are shown as forest cover for high-dense forest, cropland for low-dense forest, bare-land for bare-grounds, built-up for built-up and water bodies for water. The built-up area is responsible for heat generation and erosion, which results in enormous amounts of heat that are unwelcome for residents and the environment and destroy ecosystems. As a result, the ecosystem services coefficient for built-up and bare ground was determined to be zero [80]. Total ecosystem service value of each classified land cover was computed by dividing the value coefficient by the area of each land type [86,87].

71] ESV = ∑ (Ai × VCi) [79;71]

where ESV is the total estimated ecosystem service value of each land type, Ai is the area in ha of each kind of land cover, and VCi is the ecological value coefficient in USD/ha/year [71,79].

## 3 Results

### 3.1 LULC changes in the Atwima Nwabiagya North district from 2002 to 2022

**Fig. 3:**
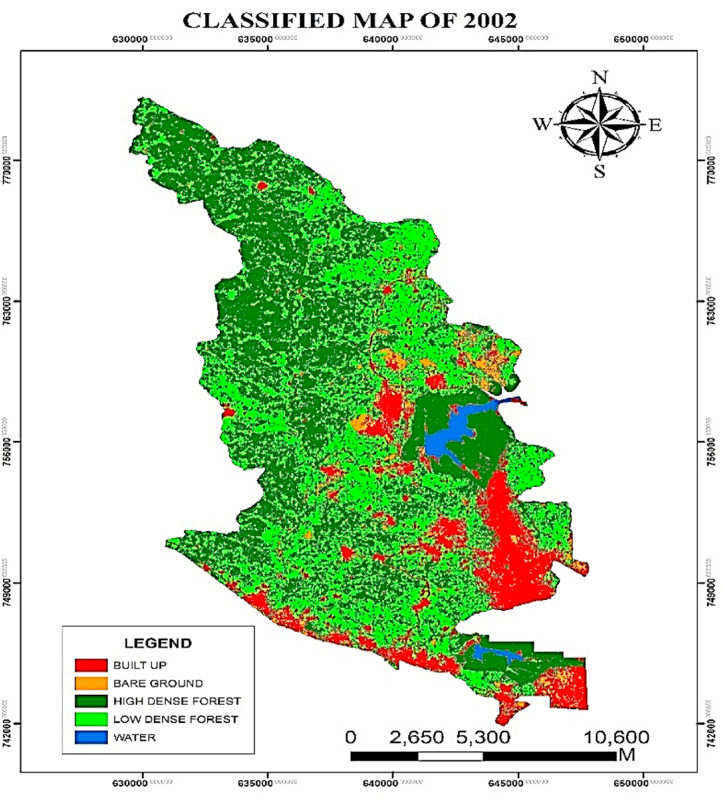
Classified map of 2002.

**Fig. 4:**
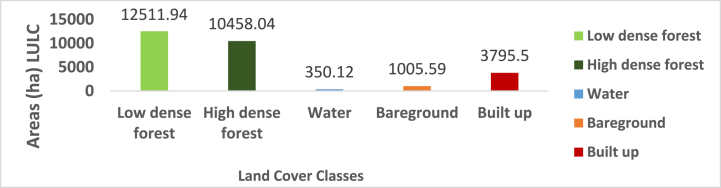
Areas (ha) LULC FOR 2002.

Based on the spatial extent of the LULC map from 2002, the Supervised Classification identified land cover classes, with low-dense forest standing out as the most significant LULC type in the Atwima Nwabiagya North District with a land surface area of 12511.94 ha. As a result, it accounted for 44.49 % of the district's total land area. According to Fig. 3, Fig. 4, the second most prevalent LULC type, high-dense forest, covered 10458.04 ha (37.19 %) of the Atwima Nwa biagya North District. The identified pixels were made up of 350.12 ha (13.50 %) of water area, 3795.50 ha (3.58 %) of built-up area, and 1005.59 ha (1.26 %) of bare ground.

**Fig. 5:**
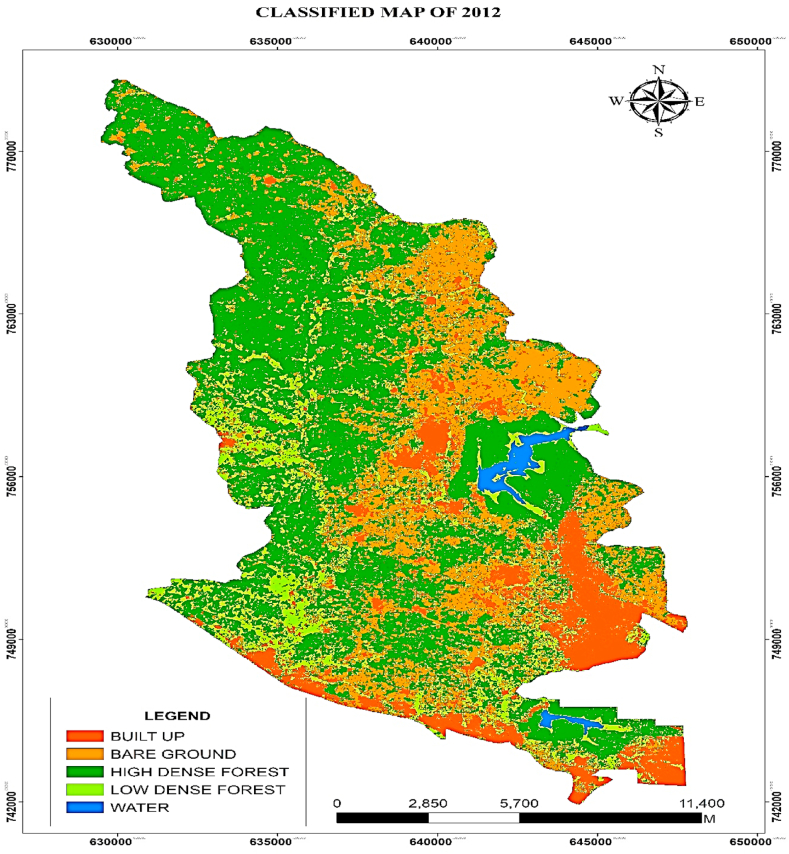
Classified map of 2012.

**Fig. 6:**
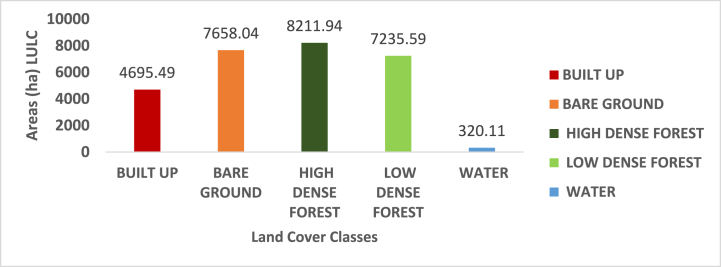
Areas (ha) LULC for 2012.

Based on the identified pixels, it was found that in 2012, the land areas of the two most important LULC classes (high-dense and low-dense forests) that had dominated the study area in the year before (2002) had decreased. Low-dense forest representing the largest dominating LULC type in the study area in 2002 had declined to 7235.59ha representing 25.73 %, high-dense forest declined to 8211.94ha representing 29.2 %, Water area had reduced to 320.11ha (1.13 %) whilst Built-up and Bare-grounds had stood and increased at 4695.49 ha (16.69 %), 7658.039ha (27.23 %) respectively as indicated in (Fig. 5, Fig. 6).

**Fig. 7:**
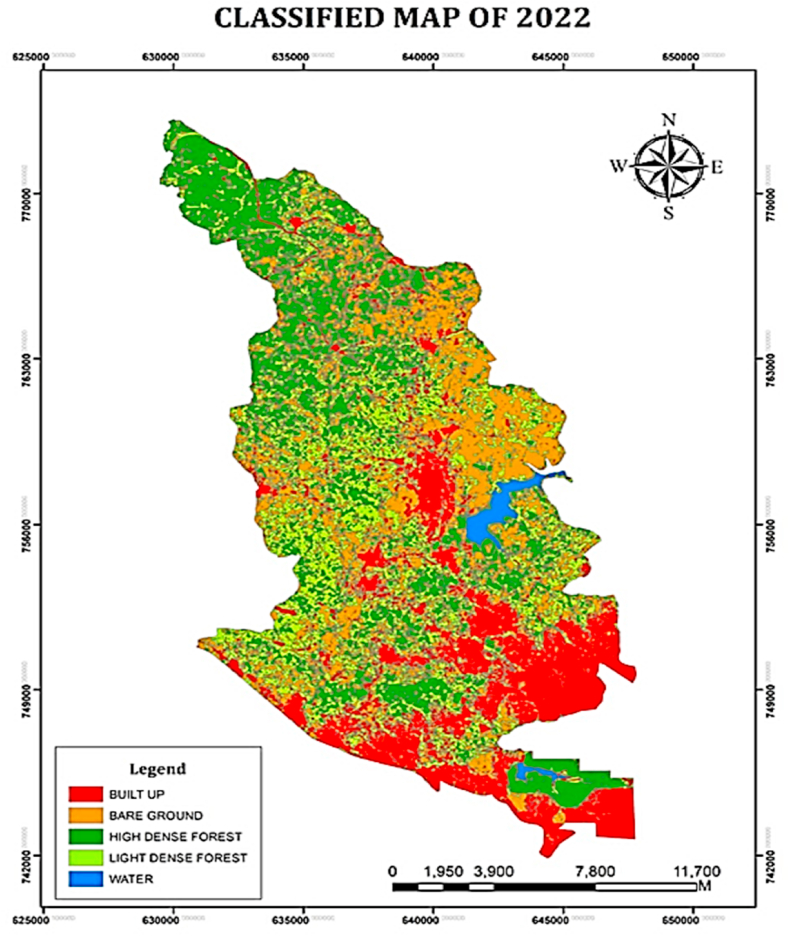
Classified map of 2022.

**Fig. 8:**
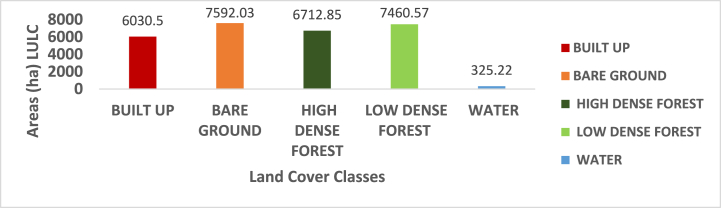
Areas (ha) LULC for 2022.

The assessment of Landsat 8 2022 satellite imagery indicated that built-up and bare-grounds land areas have increased in the Atwima Nwabiagya North District. Built-up and bare-ground covered 6030.5 ha (21.44 %) and 7592.03ha (27 %) of the land surface area respectively. This shows that the surface area of built-up and bare-grounds have increased from 2002, 2012 and 2022. Following the classification, it was found that both high- and low-dense forests lost land surface area to bare ground and built-up areas. Fig. 7, Fig. 8 show that the area of water has slightly grown to 325.22 ha representing 1.16 % while the area of high dense forest has decreased to 6712.85 ha representing 23.87 % and the area of low dense forest has decreased to 7460.57 ha representing 26.53 %.

#### 3.1.1 LULC change within the Atwima Nwabiagya North District

**Fig. 9:**
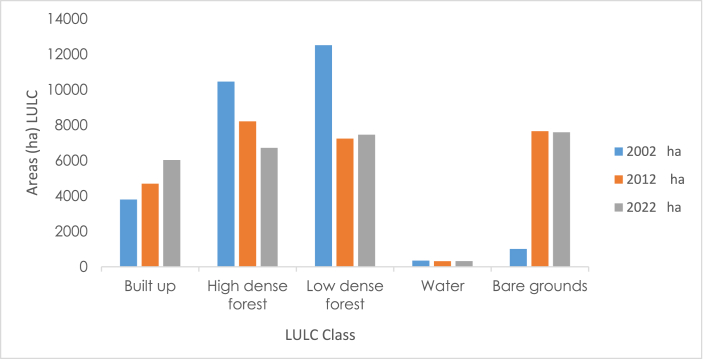
Shows the LULC proportions for 2002, 2012, and 2022 in graphical form.

The LULC maps and analysis demonstrate that over the course of the 20-year study period from 2002 to 2022, a number of changes have taken place in the study area. Fig. 9 shows a graphical representation of LULC proportions in the years 2002, 2012, and 2022 while Table 3 shows the LULC proportions in those years. The majority of the LULC changes over the 20-year study period happened in bare grounds, built-up areas, high-dense forests, and low-dense forests, whereas the water area underwent relatively little changes.

**Table 3:** LULC changes in the Atwima Nwabiagya North District after the classification (Area (ha), Percentage (%)). (LULC PROPORTION).

LULC Classes	2002 ha	2012 ha	2022 ha	2002%	2012%	2022%
Built up	3795.50	4695.49	6030.50	13.50	16.69	21.44
High dense forest	10458.02	8211.94	6712.85	37.19	29.2	23.87
Low dense forest	12511.94	7235.59	7460.57	44.49	25.73	26.53
Water	350.12	320.11	325.22	1.26	1.13	1.16
Bare grounds	1005.59	7658.04	7592.03	3.58	27.23	27
Total Area	**28121.17**	**28121.17**	**28121.17**	**100**	**100**	**100**

#### 3.1.2 LULC change patterns in the Atwima Nwabiagya North District from 2002 to 2022

**Fig. 10:**
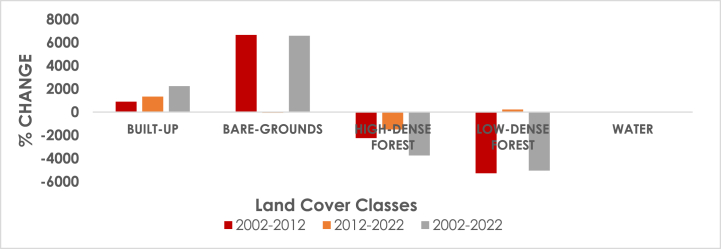
LULC trends, 2002 to 2022.

As shown in Table 4 and Fig. 10 below, the Atwima Nwabiagya North District LULC change trend study shows a change in the size of the five LULC classes throughout the 20-year period. Following the classification, it was found that high-dense and low-dense forests had the largest negative change, whilst built-up regions had the greatest positive change. Land areas with built-up and bare ground increased between 2002 and 2012, whereas areas with high-dense forest, low-dense forest, and water decreased. Positive changes were observed between 2012 and 2022 in built-up, water, and low-density forest, while negative changes were observed in high-dense forest and bare ground. Between 2002 and 2022, the land area of built-up and bare ground areas increased, whilst the land area of high-dense forest, low-dense forest, and water decreased.

**Table 4:** Land and Land cover change trend, 2002 to 2022.

LULC CLASSES	Change (Hectares) ha	ha	ha	PERCENTAGE %	%	%
Duration	2002–2012	2012–2022	2002–2022	2002–2012	2012–2022	2002–2022
Built-up	900	1335.01	2235.01	3.2	4.7	7.95
Bare-grounds	6652.45	−66.01	6586.44	23	−0.23	23.42
High-dense forest	−2246.1	−1498.38	−3744.46	−7.99	−5.33	−13.32
Low-dense forest	−5276.35	224.98	−5051.37	−18.76	0.80	−17.96
Water	−30.01	5.11	−24.9	−0.11	0.02	−0.09

#### 3.1.3 LULC changes detection from 2002 to 2022

**Fig. 11:**
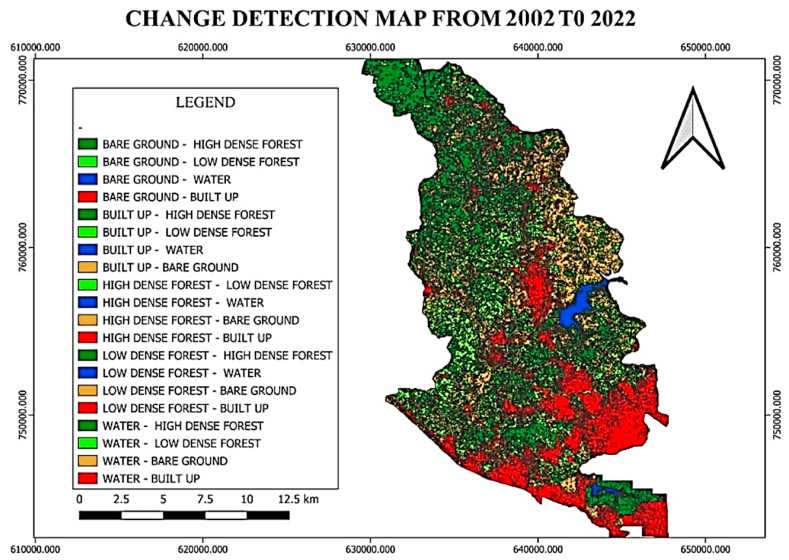
LULC change detection (2002–2022).

According to the land cover change matrix for the period 2002–2022, there will be relative changes, with low dense forest areas being replaced by built-up, bare ground, and water. Additionally, high-dense forest lost land areas to low-dense forest, bare ground, built-up , and water. Built-up, bare-ground, and low-dense forest also develops in the water areas (see Fig. 11 and Table 5).

**Table 5:** LULC change detection (2002–2022).

2022							
**2002**	LULC Classes	Built-up	Bare-grounds	High-Dense Forest	Low-dense forest	Water	Total Areas
	Built-up	**416.71**	160.97	37.79	71.11	19.20	**705.78**
	Bare-grounds	2940.99	**1412.14**	559.44	1124.75	15.09	**6052.41**
	High-dense forest	699.36	2834.86	**4293.78**	3488.43	23.63	**11340.06**
	Low-dense forest	1933.02	3229.37	1798.42	**2737.99**	15.11	**9713.91**
	Water	15.96	14.02	9.38	18.48	**251.17**	**309.01**
	Total Areas	**6006.04**	**7651.36**	**6698.81**	**7440.7**	**324.2**	**28121.17**

#### 3.1.4 LULC classification accuracy

For various LULC classes for 2022, 2012, and 2002, the overall classification accuracy, Kappa coefficient, user, and producer accuracies were determined and displayed in Table 6, Table 7, Table 8, respectively. While the LULC maps for the years 2012 and 2002 had overall accuracies of 96.00 % and 95.00 %, respectively, and with Kappa coefficients of 0.9505 and 0.9375, the LULC map for the year 2022 had an overall accuracy of 99.00 %. These results fall within the permitted range, therefore the study moved forward and used the classification output to estimate the forest ecosystems inside each of the study area's several LULC classes.

**Table 6:** Accuracy Assessment for classified map 2022.

Reference Data					
Class Name	Reference Totals	Classified Totals	Number of Corrects	Producers Accuracy	Users Accuracy
Built-up	20	20	20	100 %	100 %
Bare-ground	20	20	20	100 %	100 %
High-dense forest	20	19	19	95 %	100 %
Low-dense forest	20	21	20	100 %	95.24 %
Water	20	20	20	100 %	100 %
Total	100	100	99		
Overall Accuracy	99.00 %				
Overall Kappa	0.9875

**Table 7:** Accuracy Assessment for classified map 2012.

Reference Data					
Class Name	Reference Totals	Classified Totals	Number of Corrects	Producers Accuracy	Users Accuracy
Built-up	20	17	17	85 %	100 %
Bare-ground	20	24	20	100 %	83.33 %
High-dense forest	20	19	19	95 %	100 %
Low-dense forest	20	20	20	100 %	100 %
Water	20	20	20	100 %	100 %
Total	100	100	96		
Overall Accuracy	96.00 %				
Overall Kappa	0.9505

**Table 8:** Accuracy Assessment for classified map 2002.

Reference Data					
Class Name	Reference Totals	Classified Totals	Number of Corrects	Producers Accuracy	Users Accuracy
Built-up	20	20	20	100 %	100 %
Bare-ground	20	23	20	100 %	86.96 %
High-dense forest	20	22	20	100 %	90.91 %
Low-dense forest	20	18	18	90 %	100 %
Water	20	17	17	85 %	100 %
Total	100	100	95		
Overall Accuracy	95.00 %				
Overall Kappa	0.9375				

### 3.2 Current state of forest ecosystem service values

For this study, forest ecosystems value for Atwima Nwabiagya North District is calculated using values derived by Refs. [79,80] and modified by Ref. [82]. It was noted that the value of ecosystem services varied in the study area from the study period (2002–2022). While built-up and bare-grounds regions have risen, the value of the forest ecosystem as a whole has decreased (see Table 9). These are the major factors contributed to the changes of forest ecosystem services value in the Atwima Nwabiagya North District. For the year 2002, the ecosystem service value for high-dense forest, low-dense forest and water were USD 22.68, USD 8.75 and USD 2.30 respectively (see Table 9). In 2012, ecosystem service values for high-dense forest reduced to USD 17.81, low-dense forest reduced to USD 5.06 and USD 2.10 was for water. In 2022, ecosystem service values for high-dense forest further reduced to USD 14.56, low-dense forest reduced to USD 5.23 and water also reduced to USD 2.13.

**Table 9:** Ecosystem service values.

LULC Classes	2002	2012	2022
High-dense forest ($)	22,681,772.10	17,810,383.95	14,559,097.59
Low-dense forest ($)	8,750,475.48	5,060,354.58	5,217,698.84
Bare-ground ($)	0.00	0.00	0.00
Water ($)	2,294,325.86	2,097,671.23	2,131,156.90
Built-up ($)	0.00	0.00	0.00
Total ($)	**33,726,573.44**	**24,968,409.76**	**21,907,953.33**

Again, throughout the research period (2002–2022), the total value of the ecosystem decreased from USD 32.73 million to USD 21.91 million (see Table 9). It was discovered that the change in high-dense forest is the main cause of this alteration. The loss of low-dense forest in the study area was the next significant factor in the decline of the value of the entire forest ecosystem. In the research area, it has also been noted that LULC modifications have caused a total loss of water that will cost $2.29 in 2002, $2.10 in 2012, and $2.13 in 2022. Considering that the loss of waterbodies is one of the major contributors to loss of forest ecosystem service value, the reported values here might be underestimated, as the spatial resolution of the Landsat images used for the study could not capture the loss of small water bodies, such as springs [73]. LULC changes and associated loss of forest ecosystem services value are expected to increase in the near future if high-dense forest, low-dense forest and water continue to decline. Other studies have also shown similar results where loss of forest covers are major contributors to the reduction of ecosystem services value [71,79,81,and^88^]]. The expansion of bare-grounds in the district could also contribute significantly to these changes.

### 3.3 Impacts of LULC changes to forest ecosystem service values

Analysis of the spatial extent of LULC changes in section 3.0 discovered a decline of forest cover (high and low dense forests) of the study area. From the study, 699.36 ha of high-dense forest and 1933.02 ha of low-dense forest have lost to built-up (see Table 6). A related study by Kullo et al. [34], revealed similar results where 4178.5 ha (7 %) of closed and open forests have lost to crop land and settlement. Antwi-Agyei et al. [5], showed that since 1970, 24.6 % of high-dense forests and 15.8 % of sparse forests have disappeared within the district. The extensive increase in built-up in the area have led to 23 % decrease in forest cover [25]. Jebiwott et al. [89], established that a reduction in forest cover as a result of LULC changes affects the ability of forest to regulate the quality, quantity and flow of water in an area. These have affected the quality of water provided by the Owabi dam in the district [16]. According to Ameyaw and Dapaah [16], water turbidity values in the Owabi dam of the district have been affected. There is also high demand of forest resources within the district giving rise to LULC changes [35].

As a result of LULC change, residents have faced a possible displacement by flooding from the confluence of the Owabi and Anomakosa rivers [90]. Debris and other undesired elements have been deposited in the Anomakosa rivers and its tributaries, creating a recipe for floods whenever it rains strongly in the study area [91]. A report by CitizenOne [92] (Self media writer, 2021) had seen that perennial flooding is becoming a normal phenomenon in the Ashanti regional capital, Kumasi. This is as a result of continuing destruction of natural habitats like high-dense forest into other land use types [31]. Urban sprawl has affected land use patterns with much of the natural physical environment of the area undergoing rapid conversions to various land uses [78]. These have resulted in the potential reduction of forest ecosystem service values and some of the ecosystem services within the district [16].

## 4 Discussion

### 4.1 LULC changes in the Atwima Nwabiagya North district from 2002 to 2022

In comparison to other estimating techniques, LULC analysis is a popular technique for determining a location's spatial and temporal variation [79]. It has been identified as one of the main drivers of change worldwide [[93], [94], [95]]. Ghana's urbanization is accelerating [96] with the majority of the urban population centered in Accra and Kumasi [5]. Population growth has been emphasized as the main driver of LULC changes, especially in developing countries [97]. According to Ghana's census data from 1984 to 2010, the population of the Kumasi Metropolitan Assembly increased at a pace of more than 5 % yearly [98], making it one of the cities with the most rapid growth in the nation [99]. As Kumasi's land cover has been rapidly changing, one of the districts that is urbanizing the fastest is the Atwima Nwabiagya North District. The land change in the Atwima Nwabiagya North from 2002 to 2022 is consistent with Ghana's economic and population growth rate, which has been well-documented to be faster since 2007 than the norm for Sub-Saharan Africa [76].

LULC analysis of the study results have seen substantial changes in land cover of the study area from 2002 to 2022, mainly through the conversion of high-dense and low-dense forests to built-up and bare-ground. High dense forest which account for 10458.02ha (37.19 %) in 2002 has reduced from 8211.94ha (29.2 %) in 2012–6712.85ha (23.87 %) in 2022. The loss of high-dense forest conformed to several local and global land change studies [[99], [100], [101], [102], [103]]. A research work by Kullo et al. [34], discovered losses of 4178.5 ha (7 %) of closed and opened forests in the district. Koranteng et al. [25], and Ayesu et al. [104], investigated similar works in the district and found a decline of forest cover and agricultural lands. This reduction can be attributed to increasing urbanization in the Kumasi Metropolitan Assembly, pushing settlements back [5]. Additionally, adjustments in socioeconomic conditions are attributed to these changes through their impact on land management practices and other varied components of farming systems, institutional settings, environmental policies, and others [105].

With regard to urbanization trend and human population in the study area, built-up area is probably going to increase, leading to an increase in demand for land for infrastructure development such as commercial, residential, and others [76]. This is evident to the rise of built-up from 4695.49ha (16.69 %) in 2012–6030.50ha (21.44 %) in 2022. According to Kullo et al. [34], because of commercial activity, population growth, and porous land tenure arrangements, there is an increase in built-up areas. This also describes an increase in urban population, which causes urban areas to spread outside of cities [106]. More vegetation is removed to make room for urbanization and cultivation as human population rises [107]. The Landsat images indicate that there were changes throughout the study area, with majority of the changes taking place close to built-up areas, demonstrating the effects of human activity on the land [108]. The quick development of the built-up has also encouraged farmers to clear the forest and expand agricultural lands (low-dense forests).

When population increases and there is an increase demand for food, additional land is required to fulfill food demand [109]. Due to this, the result of the study sees increase in low-dense forest in 2022 (7460.57ha (26.52 %)) since farmers expanded their farmlands [110]. This is in agreement with reports found in the work of Bufebo and Elias [111] where agricultural lands (low-dense forests) have expanded at the expense of forest and grazing lands.

However, since LULC change increases nitrates, phosphates (PO− 4), ammonium (NH+ 4), electrical conductivity (including sodium ion (Na+), magnesium ion (Mg2+), chlorine ion (Cl−), potassium ion (K +), calcium ion (Ca2+)), chlorophyll-a and suspended solids/turbidity and decreases dissolved oxygen (DO) in downstream rivers and estuaries [[112], [113], [114], [115]]. This will have negative effects to waterbodies. From the results, it was revealed that the dam sites (Owabi and Barekese Watersheds) referring as water for the study have reduced from 320.11ha (1.13 %) in 2012 to 325.22ha (1.16 %) in 2022. Increase impervious surfaces created by LULC changes can significantly affect watershed hydrology, sediment yield [116], reduce natural infiltration, and decrease volume of water reaching water table [117]. Bare-ground areas have also seen increasing trend from 7658.04ha (27.23 %) in 2012–7592.03ha (27 %) in 2022. The expansion of bare-grounds can be attributed to overgrazing since is one of forcing drivers of LULC changes contributing to land degradation, and expansion of bare-grounds [[118], [119], [120]].

A related study by Antwi-Agyei et al. [5], revealed increased in bare-ground areas in the Owabi catchment area within the district while the areas of waterbodies and forest cover have declined. According to a study conducted at the Qeshm Island, major section of the land area was covered by bare grounds [121]. This demonstrates that the transition strength of LULC categories varies significantly throughout both space and time, hence, site-specific analysis is necessary to identify the pattern of change and the mechanisms behind these changes [76].

### 4.2 Current state of forest ecosystem service values

Analysis of LULC for the study period has indicated that forest covers (high and low-dense forests) have declined. The study has also shown that ecosystem service values have declined. Ecosystem service value for high-dense forest has reduced from USD 22.68 million in 2002 to USD 14.56 million in 2022. Ecosystem service value for Low-dense forest has also reduced from USD 8.75 million in 2002 to USD 5.22 million in 2022. The reduction of these land covers has an adverse impact on water areas as ecosystem service value of water has also reduced. These have affected forest ecosystem service values in the study area. According to section 3.2, the total ecosystem service values has declined from USD 32.73 million in 2002 to USD 21.91 million in 2022. The reduction of forest covers (high and low-dense forests) in the study area could be the major contributor to these changes. However, the reduction of water is also another contributor to the reduction of forest ecosystem service values. The expansion of bare-grounds for the study period could also be a contributor. These have shown that LULC changes for the 20 years study period has contributed to the loss of forest ecosystem service values of the study area.

This loss of ecosystem service values brought on by LULC changes is a global issue rather than just a local or national one [122]. A study by Shrestha et al. [123], found a loss of 20.60 % ecosystem service value at the Kathmandu valley in Nepal as a result of LULC change. Belay et al. [71], investigated on similar study and discovered a decline of the net ecosystem service values by USD 9.78 to USD 106 between 1995 and 2020. Mekuria et al. [81], discovered that modifications to LULC in the Central Rift Valley, Ethiopia, have caused a total loss of US $62,110.4106 in ecosystem service values. Since LULC changes affect the structure, function and efficiency of ecosystems [124], these will affect the value of the ecosystem services [125]. If the existing patterns of high-dense forest, low-dense forest, and water continue to decline, these changes in LULC and the corresponding loss of value of forest ecosystem services are projected to rise in the near future.

4.3 Impact of LULC changes to forest ecosystems service values.

Land use patterns in combination with consumption behaviors and productive activities of people [126] have destroyed a lot of the world tropical forest [127]. LULC change analysis of the study period had seen a considerable change in LULC. This is mainly converting of high-dense and low-dense forests to built-up, water and bare-grounds (see Table 3). Built-up and bare-ground areas have seen an increasing trend (see Table 3). Research results showed that conversion of natural ecosystems into agricultural areas is rising due to direct economic advantage from disasters to the environment [[128], [129], [130]]. This was seen as low-dense forest (agricultural land) increased in 2022. Expanding of agriculture to an ever increasing population continues to eat large chunks of tropical forest each year [131]. These have altered ecosystem function and structure, changing the ecosystem service values [132].

Forest ecosystem services values calculated for the study period (2002–2022) had seen a total loss of ecosystem service value from USD 32.73 million in 2002 to USD 21.91 million in 2022 (see Table 9). Related works by Kullo et al. [34], Antwi-Agyei et al. [5], and Koranteng et al. [25], have revealed that forest covers in the study area has reduced. When humans continue to clear forests, we endanger our own quality of life, jeopardize the stability of the climate and local weather, endanger other species, and weaken the beneficial services that biological diversity provides [133]. Unplanned urban sprawl caused by human pressure is destroying nearby water bodies [134] as decrease in forest cover has huge implications for freshwater and water resources [135]. These have affected the water supply by the Owabi dam in the district [16]. Alhassan [35] had also seen similar observation where ecosystem services in the area are in high demand by different users and land use encroachers.

According to Hassan et al. [136], human interaction induced LULC is responsible to 60 % degradation in ecosystem services. These have resulted to loss of forest ecosystem service values in the study area since land encroachment to forestlands affect ecosystem architecture, functions, species geographic distributions, and ecological resilience [79]. This has resulted in damages to the ecological environment of the study area [137]. Uncontrolled urbanization and rapid urban growth due to the study area's closeness to Kumasi Metropolitan Area [5,56] have contributed to the loss of forest covers [138] and ecosystem services [35] in the district. This loss of ecosystem service values primarily due to LULC changes is consistent with findings of other studies [46,100,138]]. The primary causes to the forest ecosystem service values loss were identified as increase in built-up and bare-grounds from the LULC change analysis of the study.

Also, overdependence [35], deforestation, overgrazing, encroachment, sand winning, bush burning, and illegal harvesting of forest goods were contributing factors [5,30,34]. Poverty is one of the main drivers to the decline of forest ecosystems since deforestation is highlighted [133]. This could be because poverty is the main underlying cause of overdependence in Owabi forest resources as confirmed by Ref. [35]. Since deforestation threatens the livelihoods and cultural integrity of people that depend on forest products for future generations [139].

The study has shown that LULC changes have affected forest cover, water and ecosystem service values. A related study by Ameyaw and Dapaah [16], and Alhassan [35], had also seen a declining of ecosystem services provided by the Owabi wetland and Owabi Wildlife Sanctuary within the district. Analysis of LULC change (2002–2022) of the study area have discovered a significantly change of land covers especially converting of high and low dense forests to other land use types. A related works by Kullo et al. [34], Koranteng et al. [25], Baidoo et al. [31], and Antwi-Agyei et al. [5], have discovered a decline of forest covers within the district. These show that there is a positive relationship between LULC changes and loss of forest ecosystem service values in the Atwima Nwabiagya North District. It is becoming increasingly important to understand how LULC changes impact forest ecosystems in order to convey to politicians, land planners and managers, the values and advantages of effective land management [140]. These will help the nation monitor its natural resources and implement sustainable development policies to ensure the continued health of its ecosystem services [141]. This necessitates ecological and natural resource management that is adaptive [142], which in turn creates the groundwork for bringing together many stakeholders to accommodate differing viewpoints and interests to ensure better management of land resources for improved rural livelihoods [81].

### 4.3 Limitation

The use of satellite images especially Sentinel was a challenge to have and download from the USGS. Also, Landsat images were not easily accessible especially images before 2002. As a result of this, the study was limited to Landsat images after 2002 (2002–2022) covering a period of 20 years.

## 5 Conclusion and recommendation

This study used Landsat images, related literatures and ecological assets value table with adjusted price value [82] to evaluate LUC changes and forest ecosystems service values in the Atwima Nwabaigya North District. Between 2002 and 2022, land areas under bare-ground and built-up increased to 27 % and 21.44 %, whereas high-dense forest, low-dense forest and water areas reduced 23.87 %, 26.53 % and 1.16 % respectively. Urbanization, which is seen in the Kumasi Metropolitan Area's population rise, can be connected to these developments. Due to the need for built-up land in many districts due to population growth, the district's forest ecosystem service values have decreased. Ecosystem service value for high-dense forest reduced from USD 22.68 million in 2002 to USD 14.55 million in 2022. Ecosystem service value for low-dense forest had also declined from USD 8.75 million in 2002 to USD 5.21 million to 2022. Water areas followed same trend. Everyone would agree that clearing forests through LULC change has occasionally led to the rarity or outright extinction of numerous significant plant and animal species [143]. The key concern is that if forest cover and water quality in the district keep declining, the Atwima Nwabiagya North could see a significant loss in forest ecosystem service values. If appropriate steps are not taken to reverse it, these changes in LULC are expected to have an impact on the forest ecosystem services receive from forest covers within the district. The study's findings are crucial for formulating and implementing development strategies that encourage the extension of agricultural land to increase vegetative cover and prevent the loss of forest cover and forest ecosystem service values in the district. This can be utilized to stop forest degradation in the Atwima Nwabiagya North District by local and regional government organizations, developers, and policymakers. The results of this study will be crucial in enhancing current forest policies and laws and will also act as a roadmap for future policies of ecosystem conservation and management in Ghana and other areas of the world. It would also supplement current research on how LULC changes impact the values of forest ecosystem services in Ghana and elsewhere in the world.

## Recommendation for future research

Research needs to be carried to investigate the main drivers of degradation of forest ecosystem services values in the district to improve upon this study.

## CRediT authorship contribution statement

**Richard Baidoo:** Methodology, Writing – original draft, Writing – review & editing. **Kwame Obeng:** Writing – review & editing.

## Declaration of competing interest

The authors declare that they have no known competing financial interests or personal relationships that could have appeared to influence the work reported in this paper.
